# Lack of integrase inhibitors associated resistance mutations among HIV-1C isolates

**DOI:** 10.1186/s12967-015-0734-3

**Published:** 2015-12-01

**Authors:** Andargachew Mulu, Melanie Maier, Uwe Gerd Liebert

**Affiliations:** Institute of Virology, Faculty of Medicine, University of Leipzig, Leipzig, Germany; Department of Microbiology, College of Medicine and Health Sciences, University of Gondar, Gondar, Ethiopia

**Keywords:** Integrase, Integrase inhibitors, HIV-1 subtype C, Drug resistance, Polymorphism/mutations, Ethiopia

## Abstract

**Background:**

Although biochemical analysis of HIV-1 integrase enzyme suggested the use of integrase inhibitors (INIs) against HIV-1C, different viral subtypes may favor different mutational pathways potentially leading to varying levels of drug resistance. Thus, the aim of this study was to search for the occurrence and natural evolution of integrase polymorphisms and/or resistance mutations in HIV-1C Ethiopian clinical isolates prior to the introduction of INIs.

**Methods:**

Plasma samples from chronically infected drug naïve patients (N = 45), of whom the PR and RT sequence was determined previously, were used to generate population based sequences of HIV-1 integrase. HIV-1 subtype was determined using the REGA HIV-1 subtyping tool. Resistance mutations were interpreted according to the Stanford HIV drug resistance database (http://hivdb.stanford.edu) and the updated International Antiviral Society (IAS)-USA mutation lists. Moreover, rates of polymorphisms in the current isolates were compared with South African and global HIV-1C isolates.

**Results:**

All subjects were infected with HIV-1C concordant to the protease (PR) and reverse transcriptase (RT) regions. Neither major resistance-associated IN mutations (T66I/A/K, E92Q/G, T97A, Y143HCR, S147G, Q148H/R/K, and N155H) nor silent mutations known to change the genetic barrier were observed. Moreover, the DDE-catalytic motif (D64G/D116G/E152 K) and signature HHCC zinc-binding motifs at codon 12, 16, 40 and 43 were found to be highly conserved. However, compared to other South African subtype C isolates, the rate of polymorphism was variable at various positions.

**Conclusion:**

Although the sample size is small, the findings suggest that this drug class could be effective in Ethiopia and other southern African countries where HIV-1C is predominantly circulating. The data will contribute to define the importance of integrase polymorphism and to improve resistance interpretation algorithms in HIV-1C isolates.

## Background

HIV replication requires three viral enzymes and accessory metabolites provided by the infected cell. The viral enzymes protease (PR), reverse transcriptase (RT), and Integrase (IN), encoded by the polymerase (*pol*) gene are the main targets of current antiretroviral drugs [1]. HIV-1 IN is a 288 amino acid (aa) protein encoded by the 5′ end of the *pol* gene that folds in a multimeric form into 3 functional domains: the N-terminal domain (NTD: aa 1–49) contains an HHCC zinc binding motif which is essential to facilitate IN multimerization through its extensive contacts with adjacent catalytic core domain (CCD) monomers; the CCD (aa 50–212) contains the DDE motif of the catalytic triad D64, D116 and E152 and the viral DNA binding site; and the C-terminal domain (CTD: aa 213–288) has host DNA binding activity [2–5]. IN is responsible for chromosomal integration of the newly synthesized double strand viral DNA into the host genomic DNA [2]. This chromosomal integration is a multistep process grouped in 3 major steps. The first is the formation of the pre-integration complex (which allows entry of viral genomes into the cell nucleus). The second is 3ʹ processing which prepares both ends of the proviral DNA for integration. During this process, IN recognizes conserved sequences in the long terminal repeats promoting the removal of GT dinucleotide from the 3ʹ end, resulting in new 3′ hydroxyl ends [2]. This step occurs in the cytoplasm and involves the pre-integration complex, which consists of both viral and cellular proteins that help the pre-integration complex to migrate through nuclear pores [6]. The final step is strand transfer in which target DNA is cleaved and viral DNA is joined to the 5′ phosphate ends in the host chromosome which is most likely completed by the host DNA repair machinery [2]. These enable HIV-1 to establish a permanent genetic reservoir that can initiate new virus’s production and to replicate through cellular mitosis [4, 5].

In industrialized countries integrase strand transfer inhibitors have been shown to lead to virological suppression in both treatment naïve patient as well as treatment-experienced individuals with multidrug-resistance to other drug classes [7]. However, drug resistance to this drug class has been shown to occur both in vivo and in vitro [8, 9]. Currently, there are more than 40 substitutions specifically associated with the development of resistance to integrase inhibitors (INIs). Yet, the main mutational pathways associated with INIs resistance are limited to signature mutations at IN positions 66, 92, 143, 147, 148, and 155 [8–10]. The prevalence of INIs resistant viral strains has not yet been reported; although some studies have found that 95 % of HIV-1B-infected patients treated with this drug class were susceptible and showed viral suppression [7–9]. Even though biochemical analysis of HIV-1B and C integrase enzymes suggested the use of INIs against HIV-1C, recent studies have indicated that different viral subtypes may favor different mutational pathways potentially leading to varying levels of drug resistance among different subtypes and within the same subtype in different regions [11–13]. On top of that, IN sequence data on HIV-1C which is the most prevalent circulating clade in sub-Saharan African countries is lacking [12, 13]. So, it is worth enough to conduct specific studies on the HIV-1C subtype. Besides, an increasing number of patients in sub-Saharan African countries require alternative regimes as they fail first and second line regimes containing non-nucleoside reverse transcriptase inhibitors (NNRTIs), nucleoside reverse transcriptase inhibitors (NRTIs) and protease inhibitors (PIs) due to transmitted or secondary drug resistance mutations [14–20]. This is in line with our findings of a high rate of mutations conferring resistance to NNRTIs and NRTIs inhibitors in treatment naïve [19, 20] and treated Ethiopian patients [21]. Thus, the aim of this study was to search for the occurrence and natural evolution of integrase polymorphisms and/or integrase inhibitors (INIs) resistance mutations in HIV-1C clinical isolates of ART naïve Ethiopian patients prior to the introduction of INIs of whom the PR and RT sequence was determined previously [19].

## Methods

### Patients and samples

HIV-1C chronically infected treatment naïve patients above 18 years of age and seeking treatment at Gondar University Hospital, Northwest Ethiopia in 2008 were recruited consecutively. The inclusion and exclusion criteria and the baseline characteristics of the patients and sample collection were described before [20]. Briefly, five ml venous blood was collected in vacutainer tubes containing ethylene diamine tetraacetic acid. Baseline CD4^+^ T cell count was measured using the FACSCount flow cytometer (Becton–Dickinson, San Jose, CA, USA) following the manufacturer’s protocol. Plasma samples were separated by centrifugation and stored at **−**40 °C. RNA extraction and plasma viral load determination was made with the Abbott m2000sp automated sample preparation system using mSample preparation system RNA kit and Abbott m2000rt using Quantitative Realtime HIV-1 assay, respectively (Abbott Molecular, Des Plaines, IL, USA). The lower detection limit of the assay is 40 copies/ml.

### Amplification and sequencing

The IN region of the pol gene of the HIV-1 genome was amplified by using an in-house protocol. RNA elute was reverse transcribed to cDNA using AMV reverse transcriptase (Promega Corporation, WI, USA) by an outer primer HIVINT-Rev1 (5′TGGGATGTGTACTTCTGAACTTA3′ corresponding to positions 5192-5214) at 50 °C for 1 h. Viral cDNA was amplified by nested PCR using Phusion Hot Start High-Fidelity DNA polymerase (Finnzymes, Espoo, Finland) by outer primers HIVINT-For1 (5′AAAGGAATTGGAGGAAATGAAC3′ corresponding to positions 4167-4188) and HIVINT-Rev1 and inner primers HIVINT-For2 (5′GAAATGAACAAGTAGATAAATTAGTAAG3′ corresponding to positions 4180-4204) and HIVINT-Rev2 (5′CCTGCCATCTGTTTTCCATA3′ corresponding to position 5040–5059). Initial denaturation was done at 98 °C for 2 min followed by 40 cycles consisting of 10 s of denaturation at 98 °C and 25 s of annealing at 64 °C for the first round and at 58 °C for the second round with a 40 s extension at 72 °C for both and final extension for 5 min at 72 °C. All positions are matched to HIV-1 HXB2 (GenBank accession number K03455). The RT-PCR products which showed a clear band on agarose gel were cut-off and purified by Wizard Promega PCR clean-up system (Promega, Madison, WI, USA) according to the manufacturer’s instructions.

Purified PCR products (PCR Clean-up System, Promega) were subjected to direct sequencing of both the sense and antisense strands using Big Dye Terminator Cycle Sequencing Ready Reaction kit (Applied Biosystems Incorporated, Foster City, CA, USA). Sequencing was performed using the two inner primers which allowed a double coverage of the IN genome. After running the sequencing reaction, non-incorporated dideoxynucleoside triphosphates were removed by ethanol-sodium acetate precipitation and dried by vacuum centrifugation for 12 min. The pellet was re-suspended in 20 µl high density formamide (Applied Biosystems Incorporated, Foster City, CA, USA) for denaturation and loading on the ABI prism 310 Genetic Analyzer (Applied Biosystems). Both forward and reverse overlapping sequences were manually edited with the Geneious Basic software version 5.4 and exported as a FASTA format consensus sequences. Sequences were aligned with reference set from the Los Alamos HIV database (http://www.hiv.lanl.gov) using the Mega version 5 software (http://www.megasoftware.net) and phylogenetic inferences were performed by the Neighbour-Joining (NJ) method under Kimura’s two-parameter correction. One thousand bootstrap replicates were used to assess the phylogenetic robustness of the clusters. Moreover, the REGA HIV-1 subtyping tool was used for each sequence to determine the HIV-1 subtype.

### Drug resistance analysis

Drug resistance mutations were analysed using The Stanford University HIV drug resistance database (http://hivdb.stanford.edu) and the 2015 updated drug resistance mutations list of the International Antiviral Society (IAS-USA) [22]. Although, frequency cut-off to distinguish polymorphic from non-polymorphic positions has not been proposed amino acid changes with a prevalence of at least 3 % among treatment-naive patients were considered, as used previously [23]. The rates of polymorphisms in the current study were also compared with South African subtype C (N = 72) specific polymorphisms reported by Fish et al. [12] and with the Global HIV-1 subtype C (N = 1044) isolates (http://hivdb.stanford.edu).

### Statistical analyses

The data was analysed using to SPSS version 17 statistical packages. HIV-RNA loads were transformed to log_10_ for analysis. Data were summarized as medians and interquartile range (IQR). Non-parametric tests were performed to compare median CD4^+^ T cells count and plasma HIV-RNA levels of the different groups.

### Ethical approval

The study protocol and design including the consent procedures were approved by the University of Gondar Ethical Review Committee (RPO/55/291/00). Patients were managed following the national guideline. Written informed consent was obtained from all study subjects and documented in Research Office of the University.

## Results

From the total of 45 patients enrolled in this study, 53 % were females. The mean ± SD age of the subjects was 33 ± 1.6 years ranging from 24 to 58 years. There was no significant difference in the mean age among males and females (32.4 versus 34.5, respectively; P = 0.086). According to the WHO AIDS stage defining criteria, 15.6 % (7/45), 20 % (9/45), 55.6 % (25/45) and 8.8 % (4/45) of the patients were classified into stage one, two, three and four, respectively. Baseline median CD4^+^ T cell count was 103 cells/mm^3^ (IQR: 57.0–287.0) and the proportion of patients with CD4^+^ T cell count ≤200 and >201 cells/mm^3^ was 62 and 38 %, respectively. The mean log_10_ HIV-1 RNA level of the patients was 4.54 and did not significantly differ among patients with higher and lower CD4^+^ T cells strata (log_10_ 4.65 for >201 cells/mm^3^ versus log_10_4.4 for ≤200 cells/mm^3^). As previously reported [20] in this group of well characterized patients, two patients had NNRTI associated mutations (G190A and E138G) and one patient had a NRTI resistance-associated mutation (L210 W). A subtype C-specific polymorphism associated with NRTIs (V118I) was detected in one patient. No major drug resistance mutations in PR region were found (Table 1). However, the presence of subtype C specific polymorphisms and minor resistance mutations to protease inhibitors were detected frequently.

**Table 1:** HIV-1 genotype drug resistance profile to PRI, RTI and INIs of chronically infected HIV-1 subtype C patients

ID	Age/sex	RNA	CD4 count	Resistance to PRIs	Resistance to RTIs	Resistance to INIs
5572	28/M	5.18	20	Minor R (V11FV)	S	S
5492	30/F	5.38	27	S	S	S
5493	23/F	5.28	33	S	S	S
5790	45/M	4.18	37	S	S	S
5531	24/F	6.17	38	S	S	S
5655	29/F	3.75	48	S	S	S
5551	28/M	4.66	59	S	S	S
5642	25/F	4.01	60	S	S	S
5775	45/F	3.85	61	S	S	S
5595	25/M	4.32	66	S	S	S
5592	55/M	5.82	75	S	S	S
5717	28/F	4.21	75	S	S	S
5525	30/F	4.79	80	S	S	S
5648	35/F	5.04	85	S	S	S
5591	33/M	4.37	85	S	S	S
5582	32/M	5.43	89	S	S	S
5568	36/M	3.90	92	S	S	S
5499	35/M	5.91	97	S	S	S
5604	27/M	4.64	97	S	S	S
5517	25/F	3.83	112	S	S	S
5627	30/F	3.08	115	S	S	S
5732	36/F	5.14	118	S	S	S
5479	35/F	5.44	124	S	R–NNRTIs (E138G)	S
5546	38/F	4.33	125	S	S	S
5841	58/M	4.47	129	S	S	S
5733	32/F	4.78	146	S	S	S
5713	38/F	3.86	182	S	S	S
5681	28/M	4.38	191	S	S	S
5550	43/M	4.66	209	S	S	S
5786	27/F	3.88	211	S	S	S
5837	28/F	3.76	212	S	S	S
5768	44/M	5.91	219	S	S	S
5712	39/M	4.82	224	S	R–NNRTIs (G190A)	S
5669	40/F	3.57	227	Minor R (L10I)	S	S
5585	47/F	4.60	227	S	S	S
5596	27/F	4.8	238	S	S	S
5511	20/F	4.54	262	S	S	S
5524	42/M	3.90	269	S	S	S
5485	40/M	4.50	277	S	S	S
5690	30/M	3.30	312	S	S	S
5530	24/F	3.81	336	S	S	S
5582	34/M	5.43	366	S	S	S
5630	28/M	4.17	401	S	S	S
5511	24/F	4.54	421	S	S	S
5496	40/M	3.88	751	S	R–NRTIs (L210 W)	S

*M* Male, *F* Female, HIV RNA in log_10_ copies/ml; CD4^+^ T cell count (cells/mm^3^); *PRI* protease inhibitors, *RTI* reverse transcriptase inhibitors, *INI* integrase inhibitors, *S* susceptible, *R* resistance

**Fig. 1:**
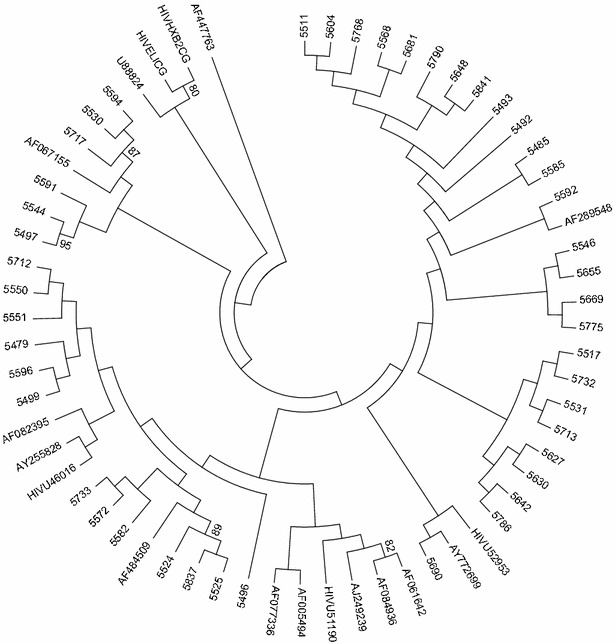
Phylogenetic relationships of the IN HIV-1C sequences (4 digit number-GenBank Accession number: KF959731-KF959775) with different HIV-1 subtype reference sequences from the Los Alamos database (http://hiv-web.lanl.gov). Bootstrap values greater than 70 % are indicated

The 45 nearly full-length nucleotide sequences of the IN region covering the first 269 amino acids (93 % of the IN gene) were with intact with open reading frames and without frameshift deletion or insertion confirming the presence of functional IN genes in all patients and primary virus isolates. However, a deletion of 6 and 12 amino acids in the catalytic core domain-CCD from codon 122 to codon 127 and from codon 188 to codon 199 was observed in two of the samples (5550 and 5713) obtained from a 43 years old male and a 38 years old female patient with baseline log_10_ HIV RNA level of 4.66 and 3.86 and CD4^+^ T cell count of 209 and 182 cells/mm^3^, respectively. Such a massive deletion has not been observed in several HIV-1C and other sequences available in HIV database. The IN intra-subtype nucleotide diversity varied between 1.7% and 5.4 % which is similar to what has been reported previously from South African subtype C IN sequences [12, 13]. All sequences were found to be HIV-1C in concordance with the corresponding PR and RT sequences [19] which is additional evidence for clade homogeneity in the region. Phylogenetic analysis of the integrase nucleotide sequences, together with viruses from the major subtypes, revealed that the majority of sequences clustered with Ethiopian HIV-1C isolates which indicates that the majority of the current Ethiopian HIV-1 epidemic descended from a single introduction into the country (Fig. 1). Clear cluster of sequences with sequences of other countries (Brazil, S. African and Indian and China) was also observed indicating that HIV-1 subtype sequences from these countries are inter-related and/or subtype C in Ethiopia may have been introduced from these countries HIV-1C lineages. None of the isolates revealed recombination.

**Fig. 2:**
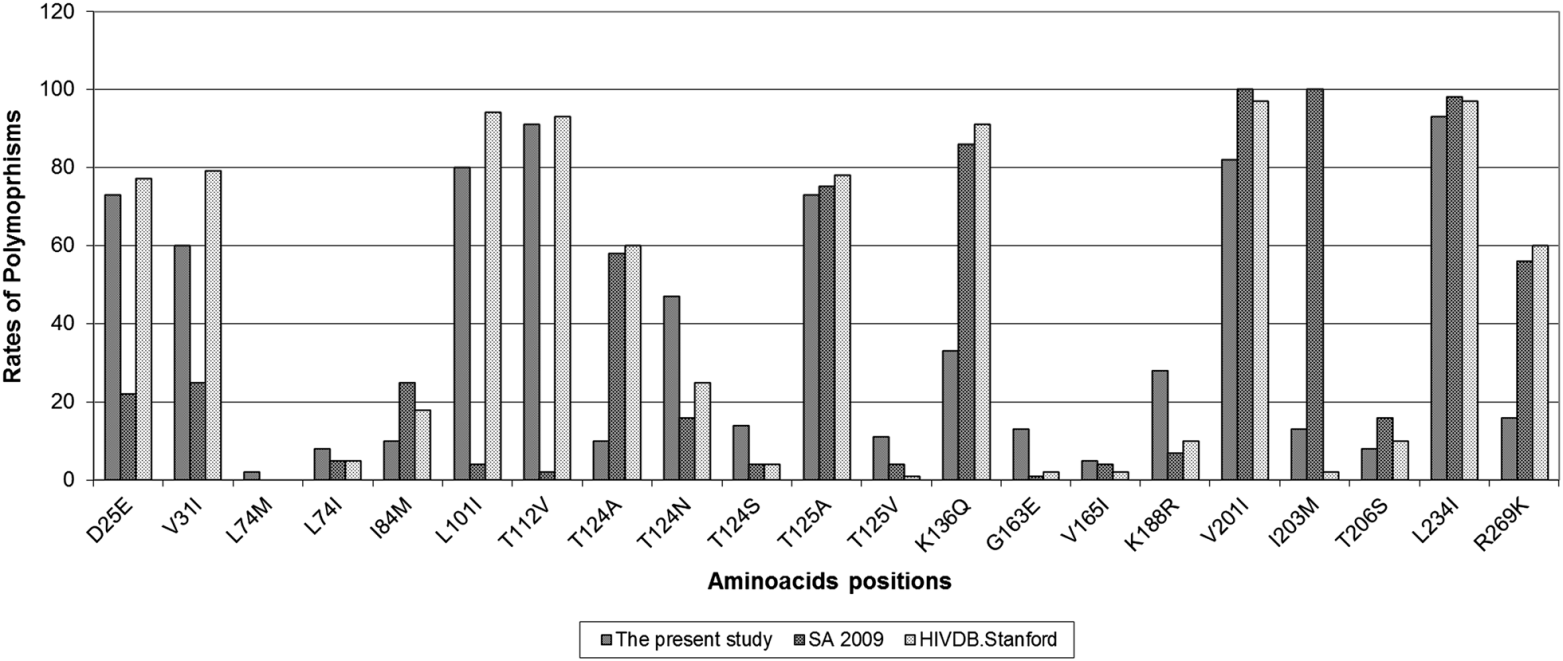
Distribution of HIV-1C IN sequences polymorphic and non-polymorphic changes of the present study (ETH 2008/2009) compared with the South African HIV-1C (SA 2009) [12] and global HIV-1C sequences (http://hivdb.stanford.edu)

Table 2 shows the frequency of the IN sequences with mutations and/or polymorphisms associated with reduced susceptibility to INIs. As expected, none of the samples contained mutations associated with primary resistance to INIs and all the isolates were found to be susceptible for INIs (Tables 1, 2). Moreover, the DDE-catalytic motif (D64G/D116G/E152 K) and signature HHCC zinc-binding motifs at codon 12, 16, 40 and 43 were found to be highly conserved. Silent mutations leading to higher increment of genetic barrier were not detected in the current study. However, polymorphic and non-polymorphic changes were observed (Table 2). Three patients (6.7 %) had minor INI-resistance mutations, i.e. L74 M and T97A. Unusual mutations at minor INI-resistance positions were observed in small number of patients (Table 2). Two polymorphic minor INI-accessory mutations (V201I and I203 M) occurred in 82.2 and 13.3 % of the patients, respectively. The rates of polymorphisms in the current study compared with South African HIV-1C specific polymorphisms [12] were similar at 6 positions, but significantly higher at 8/21 positions and significantly lower at 7/21 positions (Fig. 2).

**Table 2:** Pattern and frequency mutations and polymorphisms with potential impact on INIs reduced susceptibility of INIs among HIV-1 subtype C (n = 45)

Minor INI-resistance mutations	Frequencey
L74 M	1 (2.2)
L74I^a^	4 (8.8)
T97A	2 (4.4)
E138X^a^	4 (8.8)
G163E^a^	6 (13.3)
G163 V^a^	2 (4.4)
G163 W^a^	3 (6.6)
Minor INI accessory mutations	
V201I	37 (82.2)
I203 M	6 (13.3)

^a^Unusual mutations in non-polymorphic sites associated with minor INI mutations (according to The Stanford University HIV drug resistance database; http://hivdb.stanford.edu)

## Discussion

In this study, the IN aa sequence alignment was screened for the presence of major mutations, non-polymorphic and polymorphic changes associated with resistance to INIs (http://hivdb.stanford.edu) and additionally was categorized according to a previous report associated with INI resistance [23] (1) Major INI-resistance mutations were defined as mutations that phenotypically decrease susceptibility to raltegravir or elvitegravir by fivefold or higher and have been reported to be selected by one of these INIs in vitro or in vivo. This includes T66IAK, E92Q, F121Y, G140SA, Y143HCR, Q146P, S147G, Q148KHR, and N155HS; (2) minor INI-resistance mutations were defined as non-polymorphic or minimally polymorphic mutations that reduce INI susceptibility <fivefold by themselves or that significantly contribute to resistance when they occur in combination with other mutations (H51Y, L74 M, T97A, E138AK, S153Y, E157Q, G163RK, S230R, and R263 K); and (3) minor INI accessory mutations were defined as highly polymorphic mutations that have been reported to occur more frequently among INI-treated than INI naive patients but which have not been shown to contribute to reduced INI susceptibility (V68VI, V151IA, M154IL, V201I, I203 M, and S230 N).

The present study describes for the first time the occurrence of natural genetic polymorphisms in the IN region among HIV-1C isolates from the Horn of Africa and identifies the frequency of natural polymorphisms associated with resistance to INIs in samples from chronically infected antiretroviral drug-naïve patients. The absence of mutations associated with primary resistance to INIs and silent mutations (e.g. at codon 151) leading to higher increment of genetic barrier [24] is as expected and similar with previous reports from South Africa [11, 12], Mozambique [25], Cameron [26], Brazil [27], Quebec [28] and other parts of the world [23]. The occurrences of polymorphic minor INI-accessory mutations (L74I, T125A, V165I, T206S, L234I and V201I) are similar to previous HIV-1C isolates from South Africa [11, 12]. However, the variations on the rates of polymorphisms observed in the current study compared with South African subtype C specific polymorphisms [12] (Fig. 2) suggest the presence of intra-subtype region-specific differences.

The current study shows regional specific polymorphic and non-polymorphic changes as reported previously from southern Africa, Brazil and Quebec [28]. The absence of the non-polymorphic minor INI-resistance mutations (E157Q which could be selected by raltegravir reducing elvitegravir susceptibility) in the current study is similar with a recent report from Brazilian subtype C isolates [27] and several other studies [23, 29]. But, it was inconsistent with the findings from Quebec [28] and from South Africa [11, 12] reported in 35 and 4.1 % of the isolates, respectively suggesting subtype C regional variations involving epidemics from Ethiopia, South Africa and Quebec. On the other hand, a highly polymorphic minor INI accessory mutation (V201I) was detected in 82 % of the isolates which is significantly higher compared with the report from Cameron [26] and Quebec [28] subtype C isolates and consistent with South African isolates [11, 12]. These findings may explain the differing rates of acquisition and accumulation of mutations in subtype C isolates [30–32]. Although the significance of these residues to the current generation of INIs is not yet well known, the current findings show the need for surveillance of IN mutations and HIV-1C region specific phenotypic studies to explain the relevance of the naturally occurring polymorphisms in the absence of the signature IN mutations on susceptibility/resistance to INIs.

## Conclusion

None of the previously reported major mutations (T66AIK, E92Q, Y143RCH, S147G, Q148HRK and N155H) associated with resistance to INIs were observed in HIV-1C Ethiopian isolates, indicating that INIs can be used as a treatment option in Ethiopia and other African countries where HIV-1C is predominately circulating. However, the IN sequence variations found in the present study compared with other African subtype C isolates needs further investigation. The findings of the present study may be useful not only to explain natural IN genetic polymorphism but also to improve resistance interpretation algorithms in HIV-1C isolates about which very little is known.

Sequence data: Nucleotide sequences are deposited in National Centre for Biotechnology Information (NCBI), USA with Accession number [GenBank: KF959731-KF959775].
